# Revealing thermal effects in the electronic transport through irradiated atomic metal point contacts

**DOI:** 10.3762/bjnano.3.80

**Published:** 2012-10-24

**Authors:** Bastian Kopp, Zhiwei Yi, Daniel Benner, Fang-Qing Xie, Christian Obermair, Thomas Schimmel, Johannes Boneberg, Paul Leiderer, Elke Scheer

**Affiliations:** 1 Department of Physics, University of Konstanz, Universitätsstraße 10, 78464 Konstanz, Germany; 2 Institute of Applied Physics and DFG-Center for Functional Nanostructures, Karlsruhe Institute of Technology (KIT), Campus South, Wolfgang-Gaede-Straße 1, 76131 Karlsruhe, Germany; 3 Institute of Nanotechnology, Karlsruhe Institute of Technology (KIT), Campus North, 76027 Karlsruhe, Germany

**Keywords:** atom transistor, atomic contacts, cyclic voltammogram, electrochemically closed break junction, electronic transport, (Helmholtz) double layer, light-induced signals, temperature-induced changes, thermovoltage

## Abstract

We report on the electronic transport through nanoscopic metallic contacts under the influence of external light fields. Various processes can be of relevance here, whose underlying mechanisms can be studied by comparing different kinds of atomic contacts. For this purpose two kinds of contacts, which were established by electrochemical deposition, forming a gate-controlled quantum switch (GCQS), have been studied. We demonstrate that in these kinds of contacts thermal effects resulting from local heating due to the incident light, namely thermovoltage and the temperature dependences of the electrical resistivity and the electrochemical (Helmholtz) double layer are the most prominent effects.

## Introduction

Electronic transport on the nanoscale is one of the central topics in nanoscience. As the size of a contact between two leads is reduced to atomic dimensions, quantum phenomena become relevant in metallic point contacts [1–4], and it has even become possible to determine the conductance of individual molecules attached between two metallic tips both theoretically [4–7] and experimentally [4,8–11]. Furthermore the influence of the environment on the conductance of single-molecule junctions [12] has been revealed. In a next step towards molecular electronics, one would like to see such molecules exhibiting certain functions, e.g., the electrical current through the molecules being controllable by means of external electrodes or by light [13]. Hence studying the effect of light on nanoscopic contacts is of interest both for its own sake and for future applications.

Several theoretical investigations exist, in which the influence of light on the conductance behaviour of nanocontacts has been studied, and various mechanisms for such an influence have been suggested [14–15]. In complementary experimental studies it was shown that the conductance of electrical point contacts in a range of one to several *G*_0_ (where *G*_0_ is the conductance quantum, 2*e*^2^/*h* **≈** 77.6 µS = (12.9 kΩ)^−1^) can indeed be influenced by irradiation with light [16–18]. The observed modification in the conductivity has in this case been assigned to photo-assisted transport (PAT), partly mediated by plasmons, as the dominating mechanism. Yet, in general several additional effects are conceivable in experiments with illuminated electrical contacts, which may affect the characteristics of the contact. Although partly trivial, they can mask the intrinsic mechanisms of charge transport through the contact. For an unequivocal analysis and interpretation of the charge transport it is therefore essential to take these phenomena into account. As an obvious example, incident photons can give rise to a local increase in temperature, resulting in thermal expansion, thermovoltage, and resistance change in the leads. The effect of thermal expansion on a laser-irradiated metallic nanocontact has been demonstrated already some time ago in scanning tunnelling microscopy (STM) experiments [19–20]. Upon irradiating the STM tip with a short laser pulse, the junction resistance was observed to be drastically reduced due to the expansion of the tip, and the contact could even be switched for a short time from the tunnelling to the point-contact regime. In the following we will describe phenomena that will turn out to be related to thermal effects.

## Results and Discussion

### Electrochemically closed contacts (immersed in electrolyte) [GCQS]

The first type of sample consisted of two Au electrodes, which were immersed in an AgNO_3_/HNO_3_ electrolyte and were separated by a 50 nm wide gap. This gap was fabricated by sputtering using a carbon fibre as mask. This is the basis for the atom transistor described by Obermair and co-workers [21–23] in more detail. The contact can be repeatedly opened and closed, and well-defined conductance values can be achieved with this “gate-controlled quantum switch” (GCQS).

**Figure 1:**
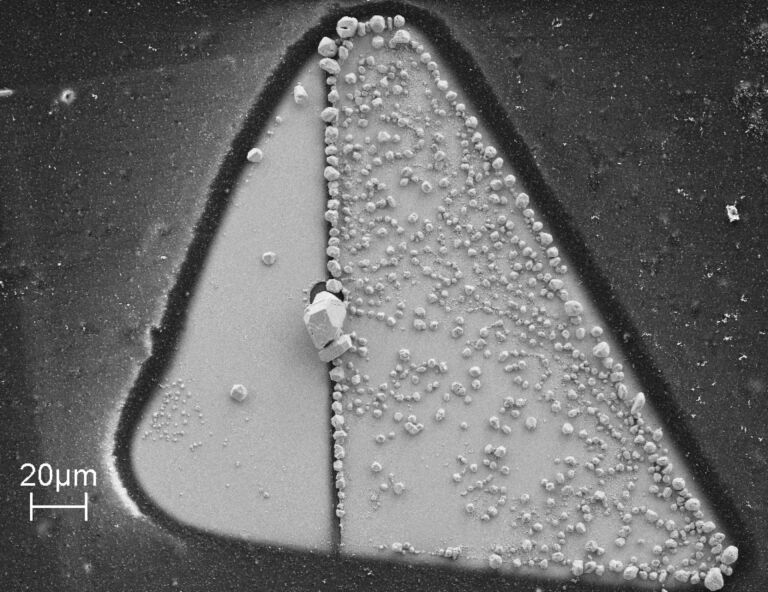
SEM image of the Au electrodes; the gap between the two segments, distinguishable by the border of the region covered by Ag crystallites, is somewhat left from the middle (size of the image 200 × 150 µm^2^).

By applying proper potentials, Ag crystallites were deposited and the contact was established. The area of the working electrodes that was exposed to the electrolyte had a triangular shape with a size of about 200 × 150 µm^2^; the remaining part of the electrodes was covered by an insulating layer of varnish. For the measurements in electrolyte, a mixture of AgNO_3_ (2 mM) and HNO_3_ (0.1 M) in bidistilled water is used. Between the two working electrodes a small potential difference of typically −12.9 mV was applied in order to determine the conductance of the contact between them. Figure 1 presents a scanning electron microscope (SEM) image after the deposition of Ag. Obviously one of the electrodes (on the right) is covered by distinctly more Ag crystallites than the other one, due to the slightly different potentials applied to the two electrodes.

**Figure 2:**
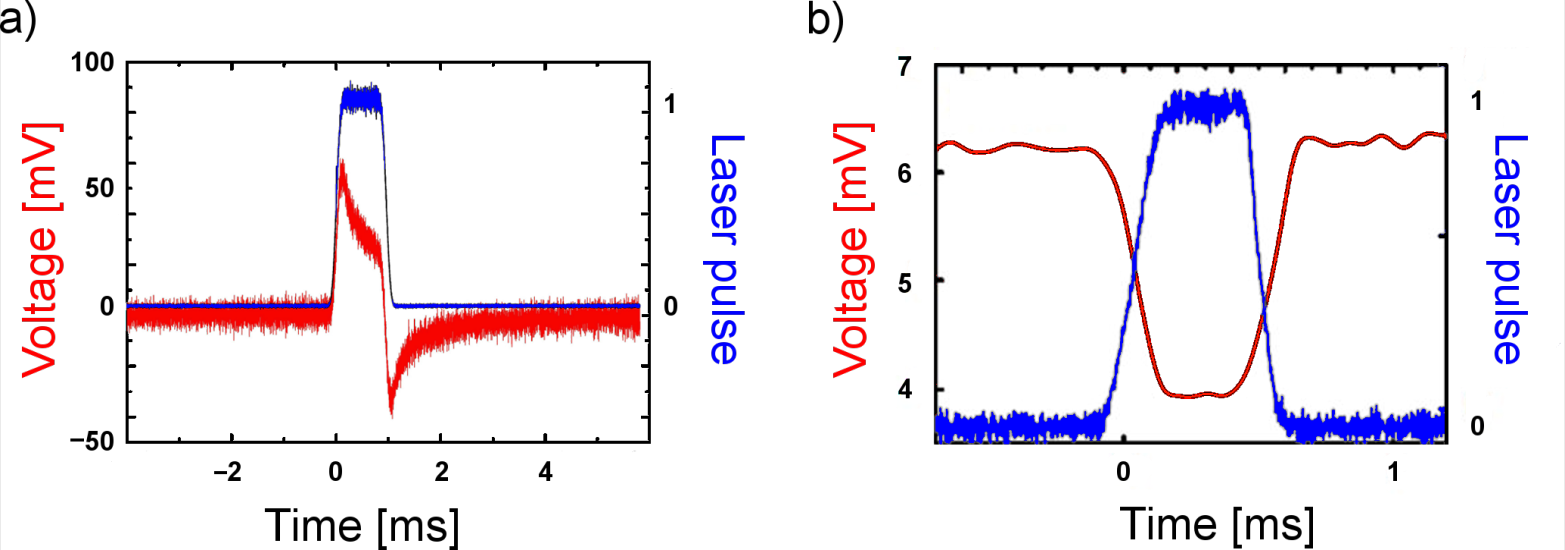
(a) Red: Light-induced signal of a gold electrode under illumination (see Figure 1) in an electrochemical environment. Blue: laser pulse (duration 1 ms). (b) Red: Light-induced signal under illumination of a mechanically controlled break-junction. Blue: laser pulse (duration 0.5 ms) [16].

The illumination experiments of these electrodes were carried out with a pulsed Nd:YAG laser (second harmonic, wavelength λ = 532 nm). The laser focus had a diameter of 10 µm, much smaller than the active electrode areas, making spatially resolved measurements feasible. A typical light-induced signal is shown in Figure 2a. Since the voltage across the contact was kept constant at −12.9 mV by the electronic circuit, this signal represents the additional current between the two electrodes induced by the light pulses. For comparison, a signal obtained in earlier measurements on a mechanically controlled break-junction (MCBJ) is shown in Figure 2b [16]. In the latter case, the signal is caused by a change in the ohmic conductance of the junction. The signal in Figure 2a, however, contains a dominating capacitive contribution.

**Figure 3:**
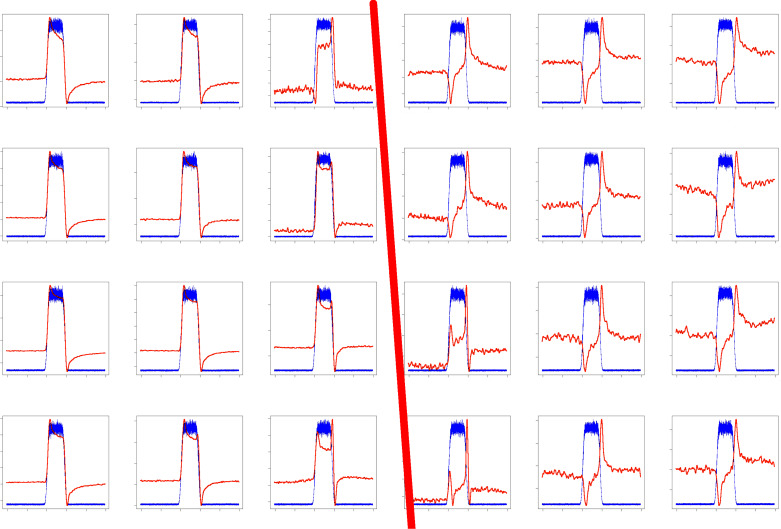
Spatial dependence of the light-induced signal (see Figure 2a) for the two working electrodes. The probed area (90 × 60 µm^2^) is split by the gap between the electrodes as the red line indicates.

A possible interpretation is that the signal for the electrolytic cell in Figure 2a does not originate from the nanoscopic ohmic contact between the two working electrodes (formed by a deposited Ag crystallite), but is rather caused by a light-induced change of the capacitance of the electrochemical (Helmholtz) double layer between the Au electrodes and the surrounding electrolyte [24]. This is corroborated by the fact that signals like in Figure 2a can also be obtained when there is no direct ohmic contact between the two electrodes, meaning that any charge transport between the working electrodes has to take place through the electrolyte. Furthermore, the signal depends on the surface conditions of the electrodes, as shown in Figure 3. Here we present a “map” of the signal for an area of 30 × 40 µm^2^ on both sides of the gap of the electrodes. The left and the right parts refer to the relatively smooth and the rough segments, respectively, as shown in the SEM image (Figure 1). Apparently, the signals are nearly constant within each segment, irrespective of the detailed position of the laser focus, but a prominent difference between the smooth and the rough electrode is observed. This supports the interpretation that the Helmholtz layer, which depends on the details of the surface of an electrode, is responsible for the signals in Figure 2a and Figure 3.

Why should the Helmholtz layer be influenced by incident light pulses? It is known that the electrical properties of the layer depend on temperature [25]. Therefore, if an incident light pulse leads to a temperature change at the metal–electrolyte interface, an electrical signal will be generated. For the laser pulses used in our experiment we estimate temperature changes at the interface in the range of a few kelvin. The corresponding signals are consistent with those reported by Gründler et al. [24] when we take into account that in our experiment only a small fraction of the metal–electrolyte interface is heated by the focused laser beam.

To be sure that what is observed here is primarily a temperature effect and not a photon-induced phenomenon, such as a photochemical reaction at the interface, we carried out a control experiment in which the Au layer was first irradiated from the electrolyte and then from the glass substrate side. If the effect is purely thermal, the light-induced signals for the two directions should essentially be the same, whereas for a photon-dominated mechanism an illumination of the Au electrode from the glass side should result in a strongly reduced signal, since the light intensity at the metal–electrolyte interface in this case is negligible. As it turned out, the signals for both illumination directions were similar, confirming the idea of a thermal effect.

All the signals reported so far were obtained for fixed potentials between the respective electrodes (reference, counter and working electrodes). By varying the potentials in a controlled way one should be able to obtain additional information about the temperature-induced changes of the Helmholtz layer. We therefore carried out voltammetric studies in order to determine the regions of the voltammogram in which the laser-induced signals are most prominent.

**Figure 4:**
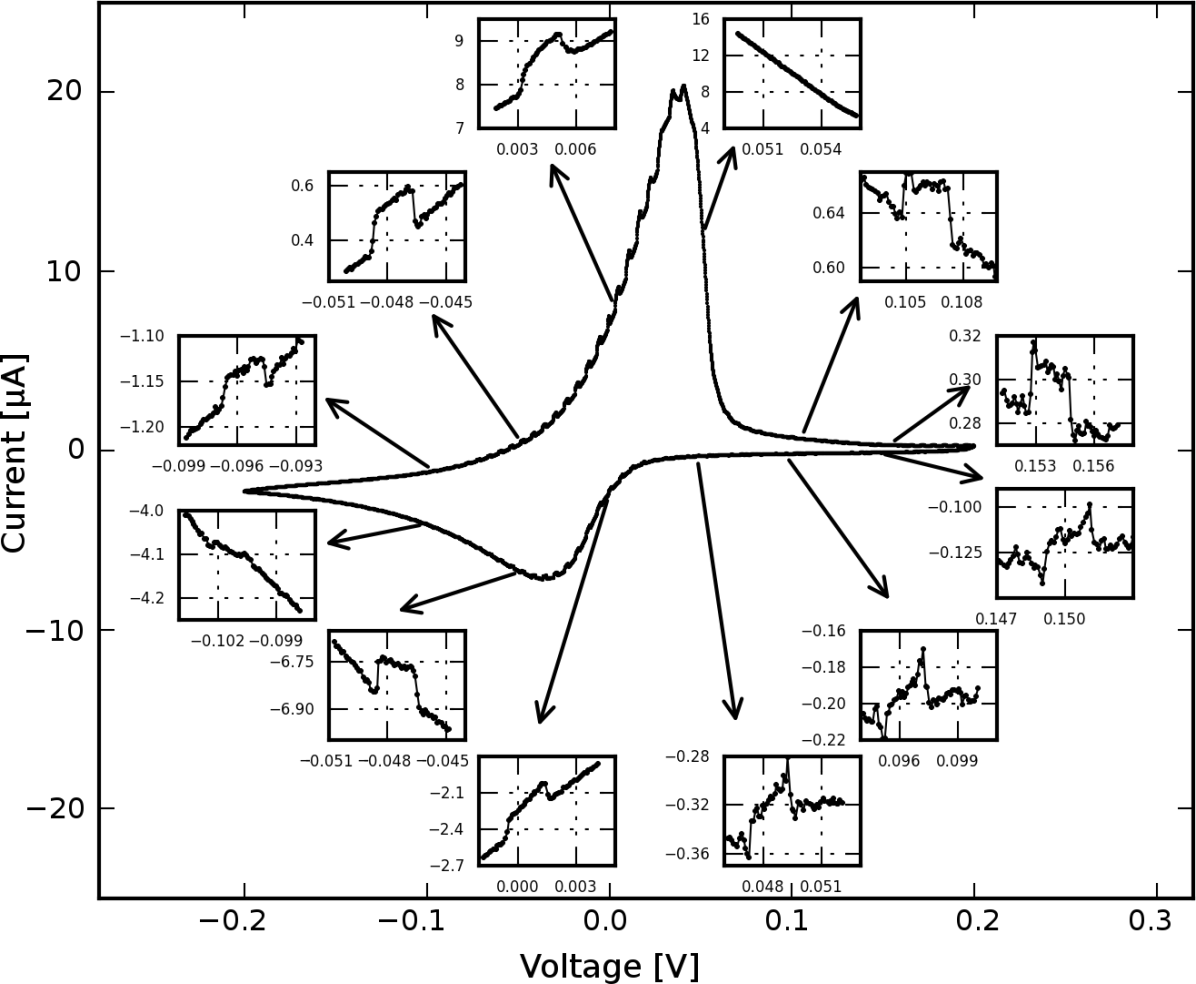
Cyclic voltammogram of AgNO_3_ under illumination. Twelve insets of zoomed areas at different potentials are presented here (scan rate is 20 mV/s).

Light-induced signals in the cyclic voltammogram of AgNO_3_ were observed as shown in Figure 4. The inset figures illustrate that the illumination triggered changes in the current: when the laser was switched on, the current jumped to a new level, and when the laser was switched off, the current went back to the original trace. The magnitude of the current increase depended on the potential and the redox states of the sample. In the forward scan, the signals were most pronounced at 0 V, with a current increase of 0.25 µA and were less pronounced at 0.1 V, where the current increase is about 0.04 µA. No light-induced signals were observed at 0.05 V. In the backward scan, the largest signals were observed around −0.05 V, with a current increase of 0.25 µA. The signals were much smaller at other potentials.

Since the light illumination unavoidably goes along with heating, it is reasonable to consider thermal effects, e.g., the temperature increase at the solid–liquid interface, as a potential origin of the light-induced signals.

**Figure 5:**
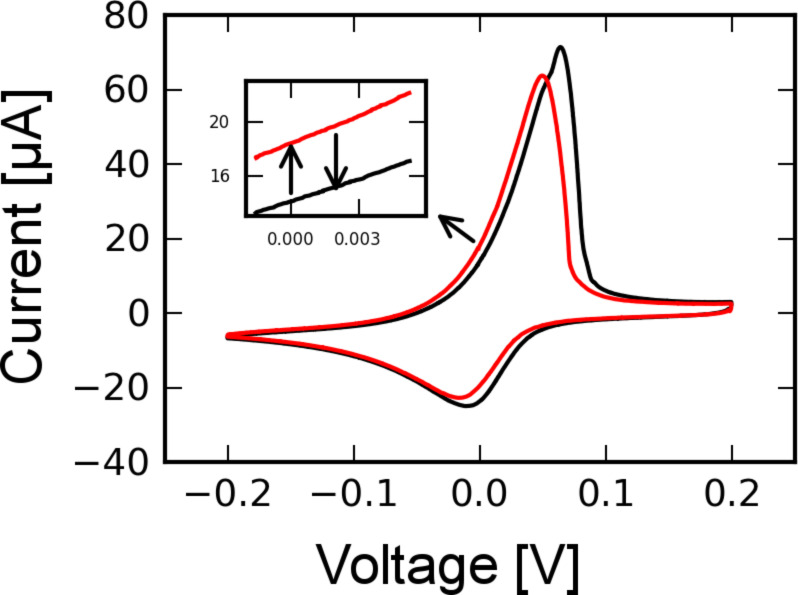
Cyclic voltammogram (CV) of AgNO_3_ at 35 °C (black) and 45 °C (red), scan rate was 50 mV/s. The temperature is increased by directly heating up the setup in a stove. The inset illustrates the current jump from the 35 °C CV trace to the 45 °C CV trace assuming that the temperature is increased quickly by light illumination.

To check this hypothesis, cyclic voltammetry was performed at different temperatures by directly heating up the whole setup in a stove. Cyclic voltammograms (CVs) recorded at 45 and 35 °C are shown in Figure 5, which indicates that the redox peak shifted to negative potentials and the peak current decreased when the temperature increased. This feature qualitatively explains the light-induced signals shown in Figure 4. When the light was switched on (indicated by the upward arrow in the inset of Figure 5), the temperature of the electrode increased and the current trace jumped to the trace corresponding to a higher temperature. As long as the electrode was illuminated the current followed the trace of higher temperature. Once the light was switched off (indicated by the downward arrow), the current jumped back to its original trace, thus forming the light-induced current changes shown in Figure 4. Furthermore, the current change in Figure 5 was most prominent at a potential of around 0 V and was less pronounced at other potentials, which also agrees with the features in the experiment on the light-induced current change (Figure 4). However, in the potential range from 0.05 to 0.1 V, light-induced signals were not observed, but current changes were observed on comparison of the CV at 45 °C with the CV at 35 °C. One reason for the discrepancy may be that the heating by light is local and the heating by a stove is homogeneous. We argue that a homogeneous temperature increase involving the whole surface area may have a more pronounced influence on the diffusion process than local heating does.

### Dried electrochemically closed contacts

**Figure 6:**
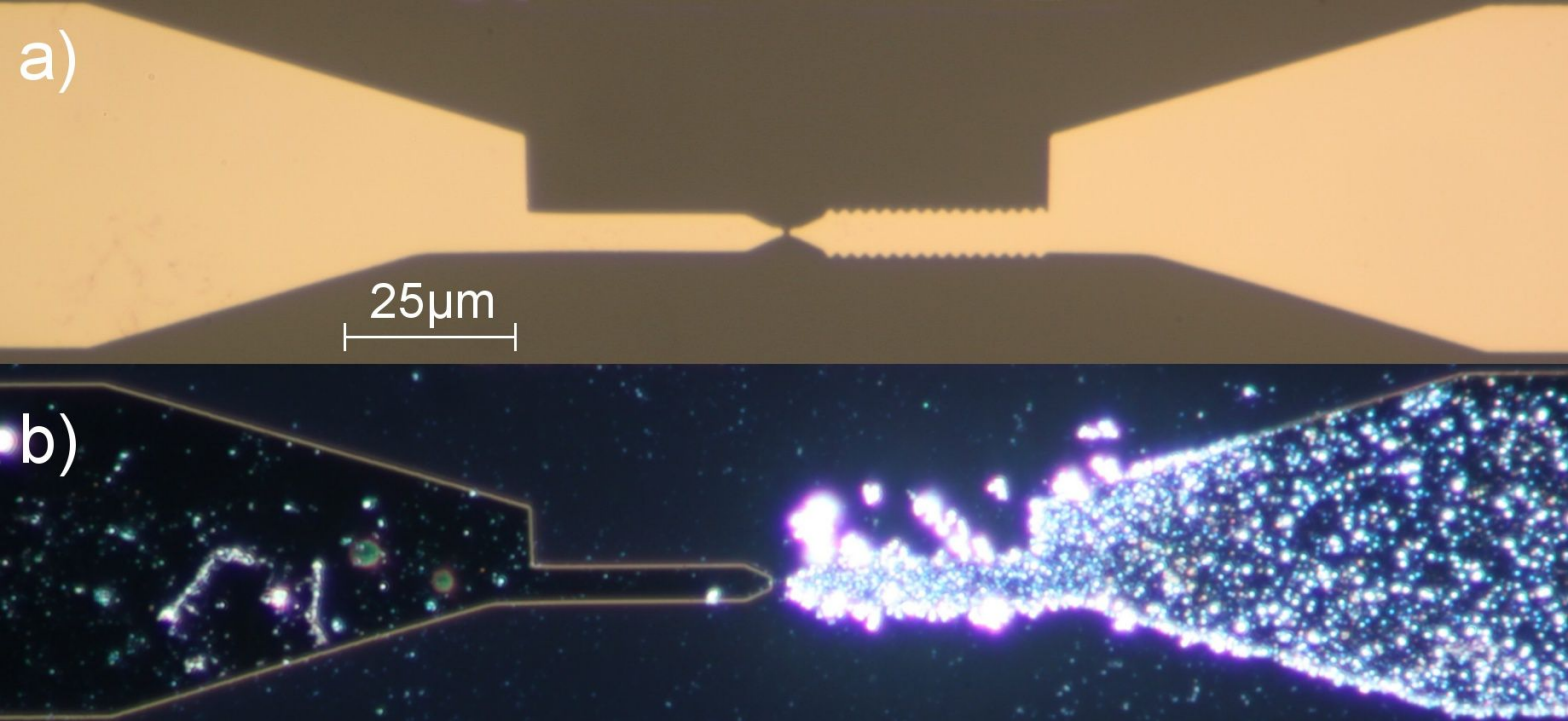
(a) Optical microscope picture of a MCBJ before electrochemical deposition of Ag (bright-field illumination). (b) Optical microscope picture after the deposition of Ag (dark-field illumination).

One possible route to eliminate the unwanted contribution of the double layer is to remove the electrolyte after the contact has been fabricated electrochemically. For these experiments we used an electrode design similar to the one typical for MCBJs (Figure 6a). The electrodes were prepared by electron-beam lithography, but in contrast to usual MCBJs we used glass substrates and a 500 nm wide gap between the two contact leads. This gap was then closed, as for the GCQS described in the previous section, by electrochemical deposition in an AgNO_3_ electrolyte. Before the electronic measurements were performed the electrolyte was carefully removed. In spite of the mechanical perturbations it was possible to keep the contact at a conductance value of a few *G*_0_, adjusted during deposition, even in the dry state. With these samples we were able to identify two sources of signals appearing upon illumination of the junction, namely thermovoltage and temperature dependence of the lead resistance.

### Thermovoltage

**Figure 7:**
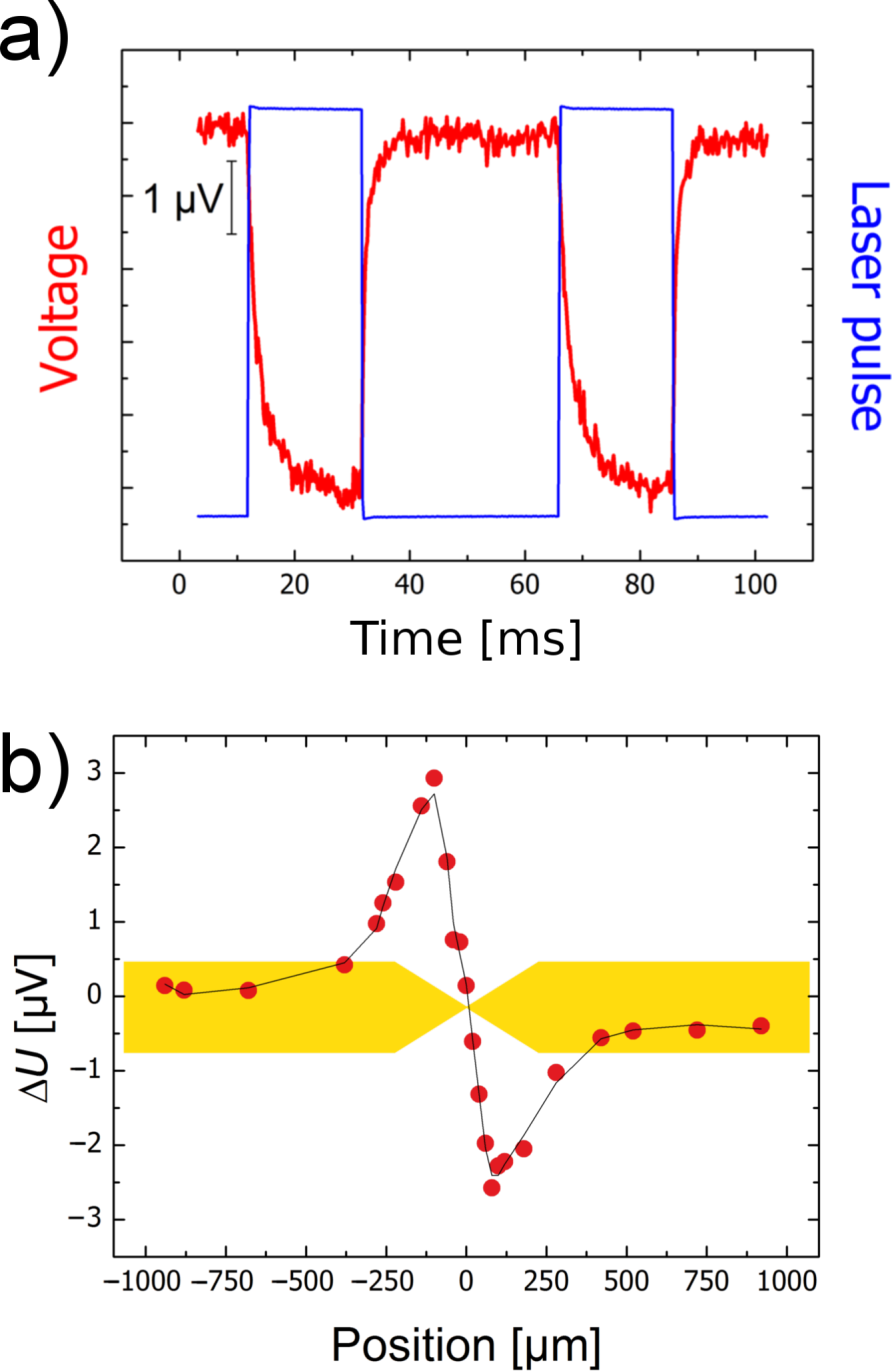
(a) Light-induced signal (red) of a dry, electrochemically closed break junction, and the laser pulse (blue). (b) Spatially resolved measurements of the light-induced signal as in (a). The signal drops to zero, when the laser focus is exactly aligned on the centre of the junction.

As an advantage compared to the GCQS in Figure 1, the location where the contact is formed is now well-defined on a sub-micrometer length scale. This allows one to determine the spatially resolved response of the junction with high resolution. As Figure 7a shows, also for such samples a light-induced signal is observable.

**Figure 8:**
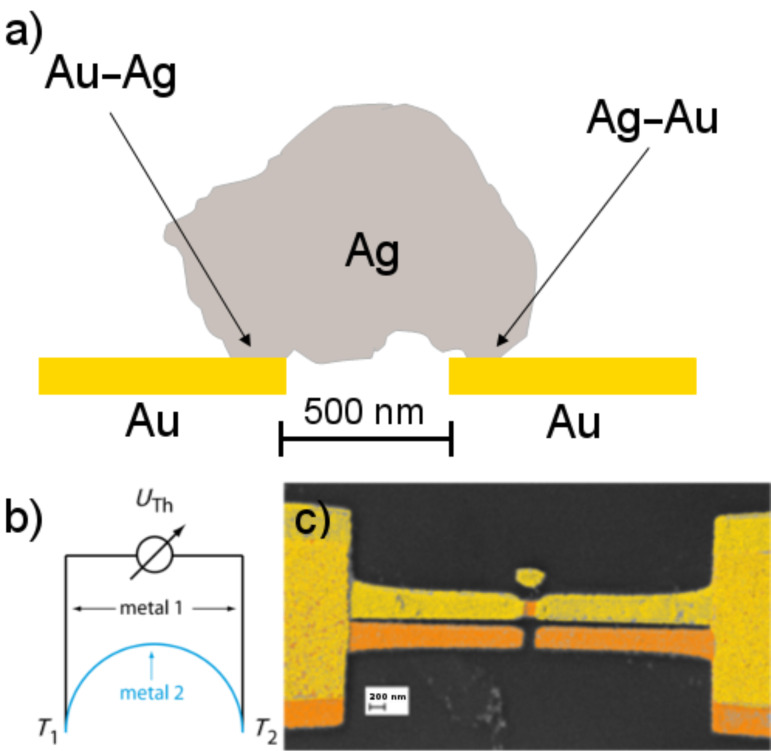
(a) Illustration of the closed contact; a silver crystallite spans the bridge across the gap between the two gold electrodes (about 500 nm). (b) Sketch of the electronic circuit of the model. (c) Coloured SEM picture of a nanothermocouple, fabricated by electron-beam lithography.

At first sight, the shape of the signal appears to be similar to the one of PAT (Figure 2b), and, as expected, a capacitive part which would result from an electrochemical double layer is not present here. Besides, the signals display a pronounced spatial dependence and are observable only in the vicinity of the point contact (Figure 7b). The signal vanishes when the laser focus is exactly at the contact position, and it changes sign when the focus is scanned from one side of the contact to the other. These observations are in contrast with the results for PAT. They can be explained when the topology of the electrochemically closed contact is taken into account, as sketched in Figure 8a.

Actually, one is not dealing with a single, but rather with two point contacts, namely an Au–Ag and an Ag–Au contact in series. These act as a thermocouple in which the sensor and the reference contact are closely spaced. For asymmetric heat input (i.e., when the junction is not illuminated exactly in the centre) one of the contacts will be at a higher temperature than the other one, resulting in a thermovoltage proportional to the temperature difference, as visualized in Figure 8b. This voltage will change its sign, when the position of the warmer contact is switched, in agreement with the behaviour shown in Figure 7b. For the combination Au–Ag, as was used here, the Seebeck coefficient is rather small, i.e., 0.3 µV/K. Nevertheless the effect is readily observable. The maximum signal in Figure 7a corresponds to a temperature difference of about 17 K between the two contacts of the junction. For other material combinations, such as Au–Pt or Au–Ni, the effect can be one to two orders of magnitude higher, and this expectation was confirmed in control experiments. Finally, we confirmed that similar effects were observed in lithographically defined junctions that were never exposed to electrolytes, Figure 8c. These control experiments demonstrate that no electrochemical process is at the origin of the observations.

### Temperature dependence of the lead resistance

**Figure 9:**
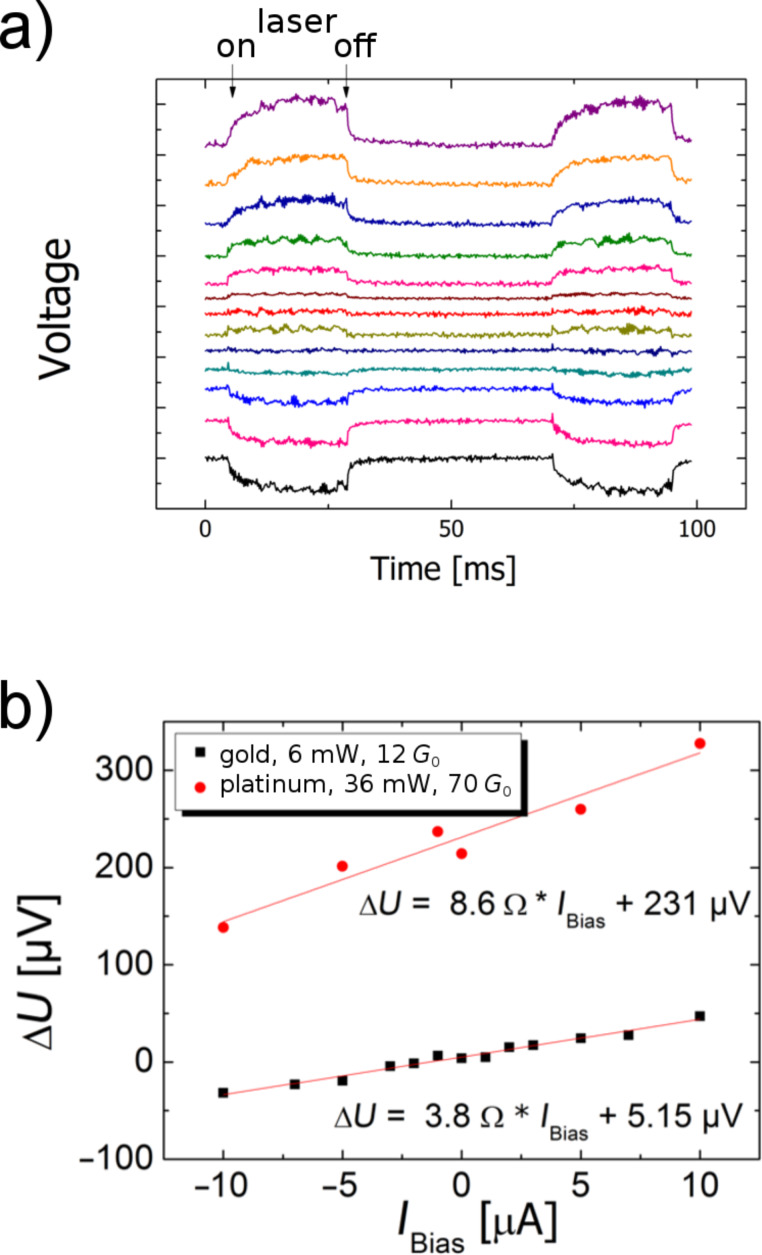
(a) Voltage change during the laser pulse at an Au–Ag–Au junction versus time for different bias currents from +10 µA to −10 µA (red curve recorded at 0 µA). One division corresponds to 50 µV. The traces are offset vertically to avoid overlap. The incident laser power during the pulses was 15 mW. (b) Voltage change versus bias current for two different material combinations, Au–Ag and Pt–Ag.

The leads towards the nanocontact, as seen in Figure 6, consist of an evaporated Au film with a thickness of 100 nm and width of 4 µm. The electrical resistance of these leads is several tens of ohms. Since the material is a pure metal, the resistance at 300 K varies roughly linearly with temperature. For a light-induced temperature change of the leads of around 10 K, as suggested on the basis of the measured thermovoltage, one will therefore expect a change in the resistance of the whole sample on the order of 1 Ω. This can also give rise to a signal upon illumination, which, however, can easily be distinguished from the thermovoltage, because it is proportional not only to the temperature change, but also to the bias current through the sample. In Figure 9 we have plotted the voltage changes across the contact upon illumination for a sequence of bias currents. As the data show, the contributions of the thermovoltage and the lead resistance to the observed signal can be separated by using the relation Δ*U = A I*_bias_* + B*, where the first term is due to the temperature-induced change in the lead resistance and the second one to the thermovoltage. The experimental values for both contributions are consistent with the estimated temperature increase during the illumination.

## Conclusion

The results show that the temperature dependence of the Helmholtz double layer is the main reason for the light-induced signal of a GCQS under laser illumination. In contrast, for the electrochemically closed, but dried contacts the thermovoltage due to a two-material system is the dominating effect. The two contacts between the two metals act as a micron-size thermocouple, which produces a thermovoltage under asymmetric laser illumination. Furthermore it was shown that a conductance change of the leads can make a noticeable contribution when small contacts are illuminated.

## Experimental

### Optical setups

**Figure 10:**
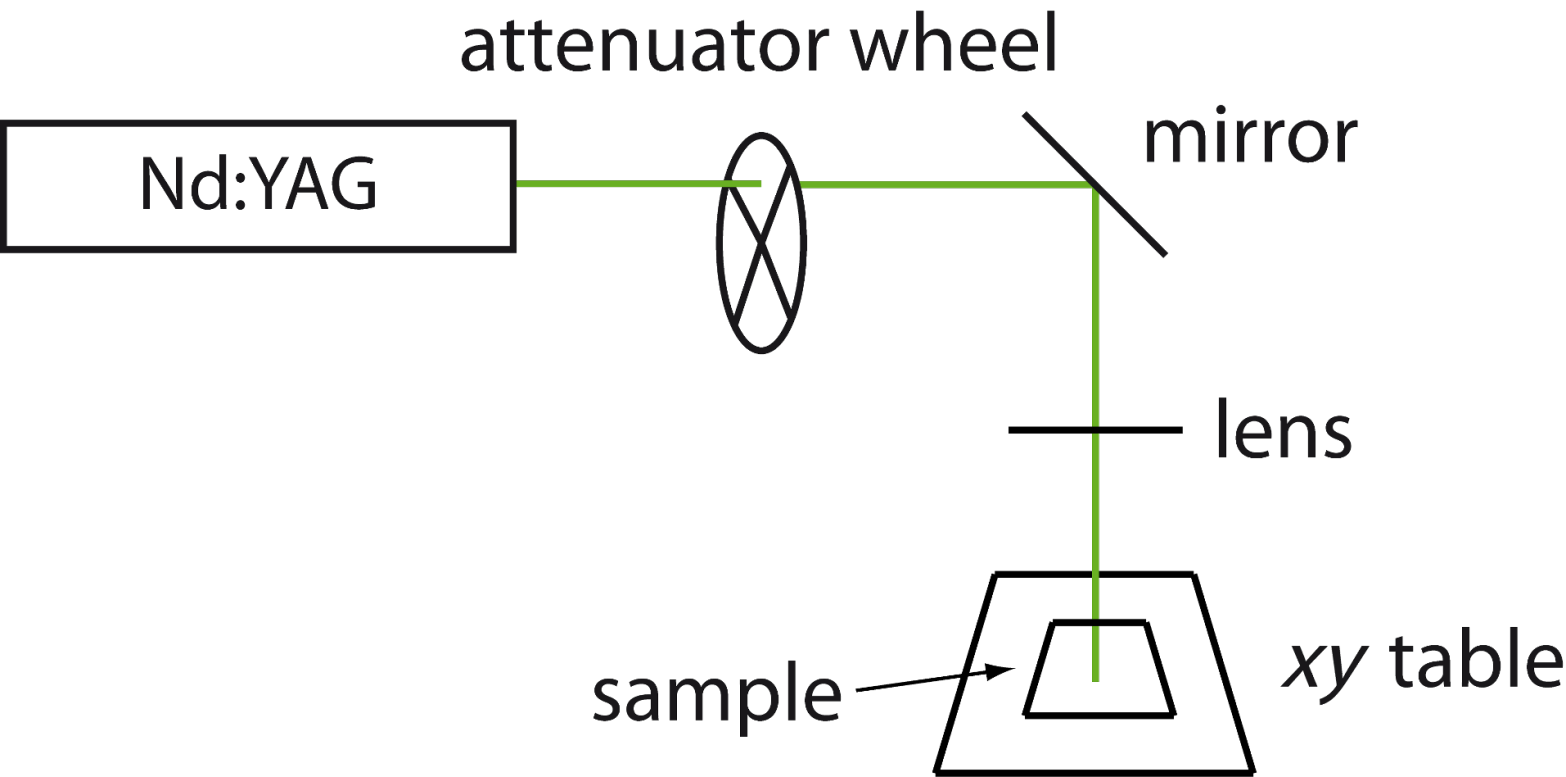
Sketch of the optical setup used for the experiments on the “dry” contacts.

For the individual experiments several optical setups with different laser sources have been used as described in the individual sections above. In all experiments we used cw-lasers, the radiation of which was chopped into pulses with a mechanical attenuation wheel. In all setups the laser beams were focussed with combinations of lenses onto the sample surface. The spot diameters are also indicated in the respective sections. In the spatially resolved experiments the samples were mounted on *xy* tables that were manually moved by using micrometer screws. As an example, we show in Figure 10 the one used for recording the data shown in Figure 7 and Figure 9.

### Electrical measurements at constant potential

**Figure 11:**
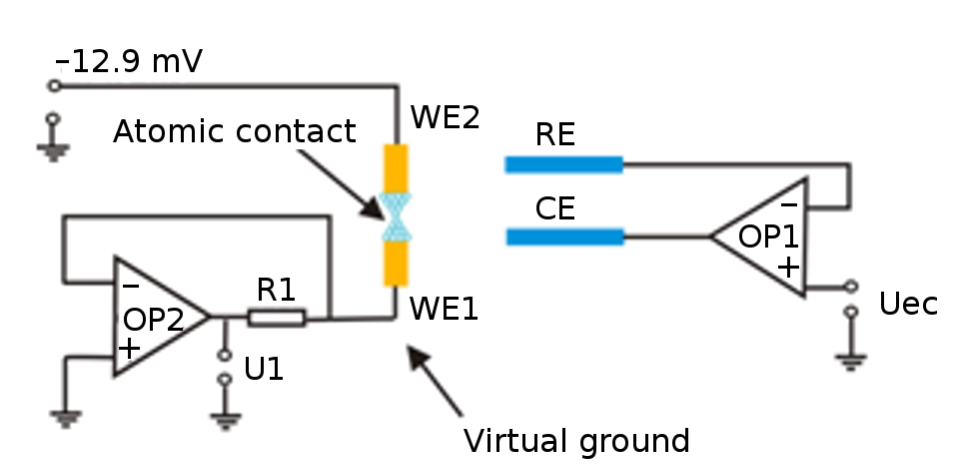
Sketch of the electronic circuit used for the measurements on the electrochemically controlled contacts. The two working electrodes (WE1, WE2) are prepared by shadow sputtering on a glass substrate. They are about 100 nm thick, and ca. 50 nm apart. The reference (RE) and counter (CE) electrodes are made of highly pure silver wires.

All electrical measurements are performed at room temperature (20–25 °C) if not stated differently. Figure 11 shows the electronic circuit for controlling the electrochemical deposition, with the two Au electrodes as working electrodes 1 and 2, and in addition a reference and a counter electrode. A voltage of –12.9 mV is applied across the two working electrodes for the conductance measurement of the metallic atomic-scale point contact. The potential at one working electrode is controlled by the virtual ground technique implemented by the operational amplifier (OP2) in this current–voltage converter. The size of the atomic contact is controlled by applying the control potential *U*_ec_ through amplifier OP1. The whole measurement is controlled by a home-written software code described in [23].

**Figure 12:**
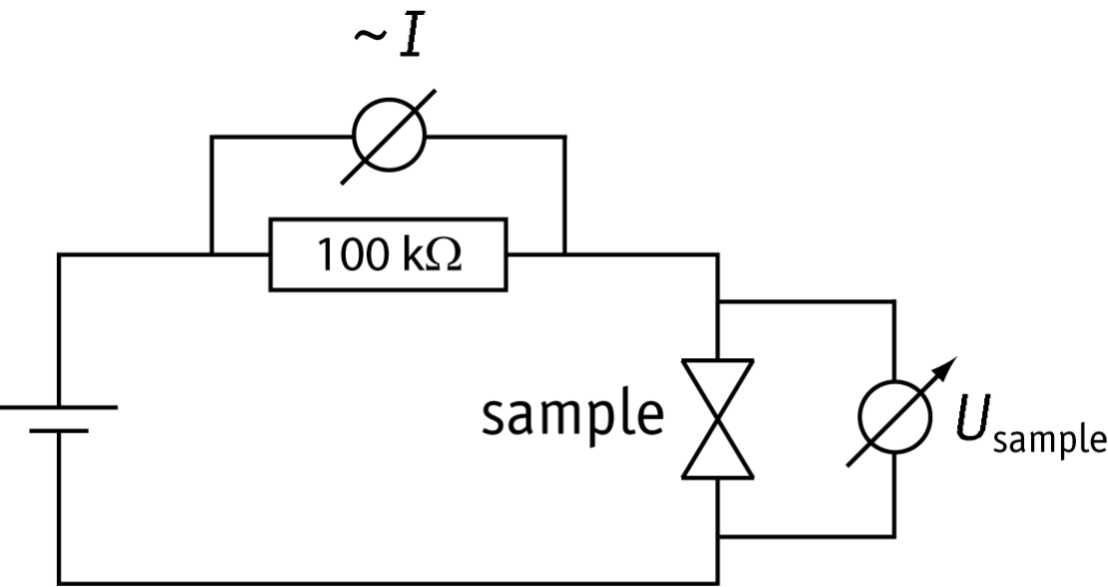
Sketch of the electrical circuit used for the measurements on the “dry” contacts.

The measurements on the dry contacts were performed in current bias mode by using a voltage source (Yokogawa model 7651) providing the voltage across the series circuit of the sample and a large series resistance of 100 kΩ (see Figure 12). The current is measured by the voltage drop across the series resistance. This signal, as well as the voltage drop *U*_sample_ across the sample are measured with fast voltage amplifiers (Femto DLPVA-100-F-D). All signals are fed to a digital storage oscilloscope (LeCroy Waverunner 6050A).

### Cyclic voltammetry

The cyclic voltammograms were recorded by using a potentiostat SP-300 (BioLogic Science Instrument), which was used to control the voltage and monitor the current. The sweep-rates are indicated in the figure captions. For recording the CVs at elevated temperature, the electrochemical cell with all the electrodes and connection cables was heated in a stove for five minutes at a preset temperature, monitored by a thermocouple to a precision of ± 2 °C. Then the cyclic voltammetry was performed in situ.

**Figure 13:**
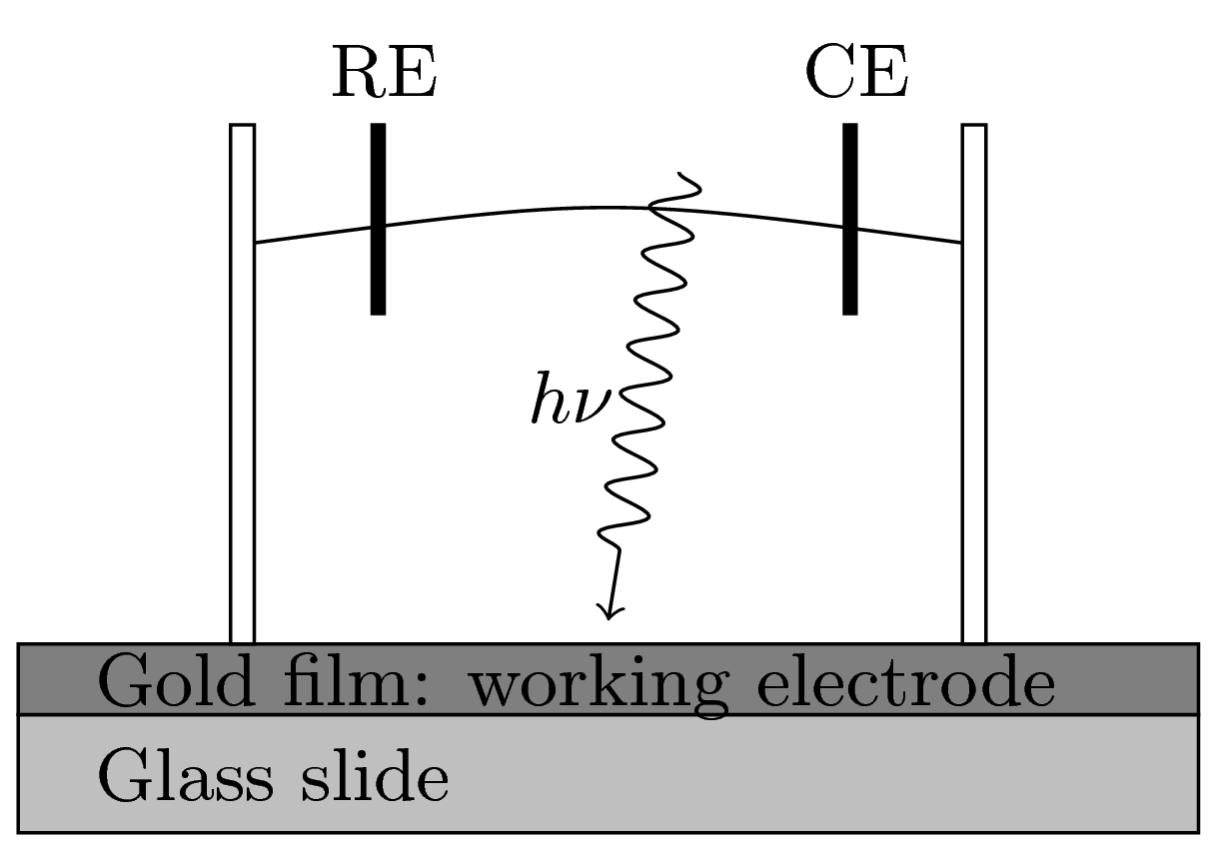
Diagram of an electrochemical cell used for studying the influence of laser illumination on the charge transport at solid–liquid interfaces.

A home-made electrochemical setup (as shown in Figure 13) was used to investigate the light-induced transport changes in the liquid environment. Two silver wires with a diameter of 0.5 mm were used as reference electrode (RE) and counter electrode (CE). A 10 nm film of titanium and 50 nm of gold was evaporated on a glass slide and used as the working electrode. Silver nitride (1 mM) in nitride acid (10 mM) was used as the supporting electrolyte. An Ar/Kr laser with output power of 5 mW and 532 nm wavelength was used as the light source. Laser pulses of 0.1 s length and 0.2 s dark time were produced by a mechanical chopper wheel, and the laser was focused to a spot with diameter of 100 μm by an optical lens.

### Electron-beam lithography

Prior to the electron-beam lithography process, a thin polyimide layer and a double layer of electron-beam resists, MMA-MAA/PMMA, were deposited by spincoating on the wafer and soft-baked in an oven at 170 °C. The polyimide layer served for both planarization of the commercial glass substrate and for enhancing the adhesion of the metal layers. To avoid deterioration of the electron-beam-defined pattern caused by charge accumulation on the insulating glass substrate, a 5 nm thin Al layer was evaporated. The electron-beam writing was performed in a scanning electron microscope equipped with a pattern generator. After being developed in a mixture of MIBK:IPA (1:3), the patterned samples were mounted in an electron-beam evaporator under high vacuum (10^−6^ mbar) and metal (Au or Pt) was deposited at a rate of 1 Å/s. The metal thickness for the electrodes to be closed by electrochemistry was in the range of 80 to 100 nm. When shadow evaporation is applied for defining the nano-thermocouple, the metal thicknesses amount to 40 nm for the first layer and 30 nm for the second layer. The lift-off is performed at room temperature in acetone for several tens of seconds. The samples are then rinsed in IPA and blown dry under a gentle flow of nitrogen.
